# RETRACTION: Risk Predictions of Hospital‐Acquired Pressure Injury in the Intensive Care Unit Based on a Machine Learning Algorithm

**DOI:** 10.1111/iwj.70256

**Published:** 2025-02-25

## Abstract

**RETRACTION:** TehranyP. M. , ZabihiM. R. , VajargahP. G. , TamimiP. , GhaderiA. , NorouzkhaniN. , MahdiabadiM. Z. , KarkhahS. , AkhoondianM. , and FarzanR. , “Risk Predictions of Hospital‐Acquired Pressure Injury in the Intensive Care Unit Based on a Machine Learning Algorithm,” International Wound Journal 20, no. 9 (2023): 3768–3775, 10.1111/iwj.14275.37312659 PMC10588304

The above article, published online on 13 June 2023, in Wiley Online Library (http://onlinelibrary.wiley.com/), has been retracted by agreement between the journal Editor in Chief, Professor Keith Harding; and John Wiley & Sons Ltd. Following an investigation by the publisher, all parties have concluded that this article was accepted solely on the basis of a compromised peer review process. The editors have therefore decided to retract the article. The authors disagree with the retraction.

**RETRACTION:** P. M. Tehrany , M. R. Zabihi , P. G. Vajargah , P. Tamimi , A. Ghaderi , N. Norouzkhani , M. Z. Mahdiabadi , S. Karkhah , M. Akhoondian , and R. Farzan , “Risk Predictions of Hospital‐Acquired Pressure Injury in the Intensive Care Unit Based on a Machine Learning Algorithm,” International Wound Journal 20, no. 9 (2023): 3768–3775, 10.1111/iwj.14275.37312659 PMC10588304

The above article, published online on 13 June 2023, in Wiley Online Library (http://onlinelibrary.wiley.com/), has been retracted by agreement between the journal Editor in Chief, Professor Keith Harding; and John Wiley & Sons Ltd. Following an investigation by the publisher, all parties have concluded that this article was accepted solely on the basis of a compromised peer review process. The editors have therefore decided to retract the article. The authors disagree with the retraction.

